# Salt, Aldosterone, and Parathyroid Hormone: What Is the Relevance for Organ Damage?

**DOI:** 10.1155/2017/4397028

**Published:** 2017-09-19

**Authors:** Cristiana Catena, Gian Luca Colussi, Gabriele Brosolo, Nicole Bertin, Marileda Novello, Andrea Palomba, Leonardo A. Sechi

**Affiliations:** Internal Medicine, Department of Medicine, University of Udine, Udine, Italy

## Abstract

Structured interventions on lifestyle have been suggested as a cost-effective strategy for prevention of cardiovascular disease. Epidemiologic studies demonstrate that dietary salt restriction effectively decreases blood pressure, but its influence on cardiovascular morbidity and mortality is still under debate. Evidence gathered from studies conducted in patients with primary aldosteronism, essential hypertension, or heart failure demonstrates that long-term exposure to elevated aldosterone results in cardiac structural and functional changes that are independent of blood pressure. Animal experiments and initial clinical studies indicate that aldosterone damages the heart only in the context of an inappropriately elevated salt status. Recent evidence suggests that aldosterone might functionally interact with the parathyroid hormone and thereby affect calcium homeostasis with important sequelae for bone mineral density and strength. The interaction between aldosterone and parathyroid hormone might have implications also for the heart. Elevated dietary salt is associated on the one hand with increased urinary calcium excretion and, on the other hand, could facilitate the interaction between aldosterone and parathyroid hormone at the cellular level. This review summarizes the evidence supporting the contribution of salt and aldosterone to cardiovascular disease and the possible cardiac and skeletal consequences of the mutual interplay between aldosterone, parathyroid hormone, and salt.

## 1. Introduction

Arterial hypertension is the most frequent modifiable cardiovascular risk factor. The NHANES (National Health and Nutrition Examination Survey) has estimated a prevalence of hypertension of 30% among the adult population, and that approximately 85% of people between 55 and 65 years will develop hypertension within their lifetime [1]. Because blood pressure control in the population is a difficult task, prevention and treatment of hypertension through interventions on patients' lifestyle have been suggested as a cost-effective strategy [2–4]. Among these interventions, a reduction of dietary salt intake could be beneficial for blood pressure control and prevention of heart failure [5].

Evidence gathered in the last decades indicates that, in addition to the well-known renal tubular actions, aldosterone regulates many cellular functions. These cellular effects of aldosterone result in the regulation of specific responses including tissue remodeling, hypertrophy, and fibrosis [6]. In fact, chronic exposure to aldosterone levels that are inappropriately elevated for the salt status causes cardiovascular damage independent of blood pressure [7]. Past animal experiments reported that chronic exposure to elevated aldosterone causes myocardial fibrosis in rats that are maintained on a high-salt diet [8] and that these changes are prevented by either administration of aldosterone antagonists or adrenalectomy [9]. In addition to these animal data, studies conducted in patients with primary aldosteronism [10] or essential hypertension [11] provided evidence that long-term exposure to inappropriately elevated aldosterone leads to a variety of organ sequelae occurring beyond what could be expected from the increase in blood pressure. Also, indirect evidence of the untoward effects of aldosterone on the cardiovascular system was obtained in clinical studies that investigated the effects of either aldosterone antagonists or adrenalectomy on patients with primary aldosteronism [10, 12].

Increasing evidence indicates that, beyond its cardiovascular effects, aldosterone excess might affect also mineral metabolism and have specific relevance for calcium homeostasis [13–15]. Because primary hyperparathyroidism is associated with poor cardiovascular outcome, a role in cardiovascular disease has been attributed also to the parathyroid hormone (PTH) [13–15]. Recent studies have demonstrated a reciprocal interaction between aldosterone and PTH, and there is growing evidence that the salt status might have a role within this interaction. This interaction between aldosterone and PTH could have clinical relevance because it could lead, on the one hand, to cardiac structural and functional changes that facilitate development and progression of heart failure and, on the other hand, to decreased bone mineral density and strength. In this narrative review, we outline the evidence supporting the contribution of salt and aldosterone to cardiovascular disease and the possible consequences of the mutual interplay between salt, aldosterone, and PTH on cardiac and skeletal damage.

## 2. The Role of Salt

The association between dietary salt consumption, hypertension, and cardiovascular disease has long been the subject of important epidemiological studies. Because of significant discrepancies among the findings of these studies, this association remains under debate [16].

### 2.1. Dietary Salt and Blood Pressure

Dietary salt consumption has long been associated with blood pressure regulation. In fact, hypertensive patients have been classified as “salt-resistant” or “salt-sensitive” depending upon their blood pressure response to an oral or intravenous salt load. Salt is distributed in the extracellular fluid and, as such, participates in blood pressure regulation [17]. The effects of salt on blood pressure, however, can be attributed to changes in extracellular volume only in part, and additional mechanisms might be involved, including changes in vascular responses to vasoactive substances and interaction with a variety of hormonal systems [18].

The relationship between dietary salt intake and blood pressure was initially investigated in the International Study of Salt and Blood Pressure (INTERSALT) population study [19]. This study demonstrated that populations with high salt intake had higher blood pressure and a greater age-related blood pressure increase than populations with low salt intake. Although prevalence of hypertension in populations with low salt consumption was unremarkable, this increased significantly after migration of these populations to geographical areas where salt intake was high. Later on, the same INTERSALT group reported a direct relationship of daily urinary sodium excretion with blood pressure in a cross-sectional investigation conducted in over 30 countries [20]. A Cochrane systematic review and meta-analysis of randomised trials concluded that decreasing daily salt intake by 4.4 grams corresponding to 1.76 grams of sodium leads to a small but statistically significant fall in blood pressure in both hypertensive and normotensive subjects [21]. In the Dietary Approaches to Stop Hypertension (DASH) study, a typical western diet was compared to a diet enriched in fruits, vegetables, and low-fat dairy products, and within each dietary group, the study subjects were assigned to eat food with different salt contents for a month [22]. In this study, a dose-response relationship between dietary salt and blood pressure levels was observed in subjects eating both the DASH diet and the typical western diet. More recently, the Prospective Urban Rural Epidemiology (PURE) study examined 102,216 adults from 667 communities all over the world to investigate the relationship between salt intake and blood pressure and to clarify whether this relationship varies across different geographical areas of the world [23]. The study countries were categorized into four different income levels, and urinary sodium excretion was estimated from a morning specimen and divided into three levels. Sodium excretion was higher in men than in women, in rural than in urban areas, and was inversely related to gross national income. This study indicated that for each 1-gram increment in urinary sodium excretion corresponding to 2.5 grams of salt, systolic and diastolic blood pressure increased by 2.1 and 0.8 mmHg, respectively. The association between urinary sodium and blood pressure had a slope that was steeper in patients with sodium excretion > 5 grams/day (corresponding to >12.5 grams of salt), in patients with hypertension, and in elderly subjects. Overall, this study indicated that the proportion of populations eating a low-sodium diet worldwide is rather small and indicated that salt intake is only weakly related to blood pressure. Thus, epidemiologic evidence indicates that dietary salt affects blood pressure, but its influence seems to be small and restricted to subjects with high salt consumption.

### 2.2. Dietary Salt and Cardiac Damage

Despite the evidence supporting the contribution of salt to blood pressure regulation, the potential benefits of dietary salt restriction on cardiovascular morbidity and mortality are uncertain. The association of daily salt intake, as assessed by urinary sodium excretion, with a composite outcome of cardiovascular events and mortality was prospectively investigated in a 3-year follow-up study [24]. An increased risk of the composite outcome was associated with dietary sodium excretion of more than 7 grams/day (corresponding to 17.5 grams of salt). Also, the risk of death and cardiovascular events considered separately was increased, and the association between dietary salt and cardiovascular outcomes was strongest among hypertensive patients. To notice, in this study, increased risk of cardiovascular events was also associated with daily sodium excretion below 3 grams/day (corresponding to 7.5 grams of salt), suggesting a J-shaped relationship. In another study by the NutriCode group, the impact of dietary salt on cardiovascular outcomes was examined by the use of a complex analysis technique [25]. Salt intake was quantified from data obtained in surveys conducted in more than 60 different countries, and the effects of salt on blood pressure and cardiovascular events were calculated with a meta-analysis. This study reported a significant dose-response relationship between salt intake and cardiovascular events and estimated that 1.65 million deaths for cardiovascular causes could be ascribed to dietary salt worldwide. However, a recent meta-analysis of seven prospective studies that compared cardiovascular mortality in patients undergoing dietary interventions to decrease salt consumption and patients on a liberal diet did not report any benefits of dietary interventions [26]. This held true also when the study subjects with normal blood pressure, hypertension, or heart failure were considered separately. In summary, although some important cumulative analyses suggest possible benefits of dietary salt restriction on cardiovascular morbidity and mortality, definitive conclusions cannot be drawn.

## 3. Aldosterone and the Heart

Landmark studies have tested the effects of aldosterone antagonists on patients with systolic cardiac failure reporting a highly significant decrease in mortality as compared to placebo [27] and supporting the view that elevated aldosterone could be harmful to the heart. Later on, studies conducted in patients with primary aldosteronism, essential hypertension, and diastolic heart failure have yielded further evidence that elevated plasma aldosterone might have untoward cardiac effects [28] and might foreshadow the onset of heart failure.

### 3.1. Primary Aldosteronism

Primary aldosteronism is associated with cardiac changes that might reflect the ability of inappropriately elevated circulating aldosterone to cause myocardial damage beyond that induced by high blood pressure itself. Longitudinal retrospective studies have shown that patients with primary aldosteronism have a greater risk of atrial fibrillation and coronary artery and cerebrovascular disease than matched patients with essential hypertension [29–32]. Also, and of greatest relevance, both surgical and medical treatments of primary aldosteronism decrease cardiovascular risk to the level of patients with essential hypertension [10, 12, 30]. Cardiac ultrasound evaluations have reported a greater increase in left ventricular mass in primary aldosteronism than in other forms of hypertensive disease [33, 34] suggesting inappropriate left ventricular hypertrophy for the hemodynamic load. In primary aldosteronism, excess ventricular hypertrophy occurs in conjunction with an abnormal pattern of ventricular filling indicating diastolic dysfunction [35]. The cardiac findings obtained in patients with primary aldosteronism were corroborated by the demonstration that also patients with familial hyperaldosteronism type 1 who have normal blood pressure and elevated aldosterone have increased left ventricular wall thickness and diastolic dysfunction in comparison to matched normotensive patients [36]. Long-term observation after treatment of primary aldosteronism showed that both adrenalectomy and spironolactone caused a significant decrease in ventricular mass [34, 37] and that the extent of this decrease was directly related to pretreatment plasma aldosterone levels [38].

### 3.2. Essential Hypertension and Left Ventricular Diastolic Dysfunction

Because of the relevance of left ventricular hypertrophy and diastolic dysfunction in patients with essential hypertension, the possible contribution of circulating aldosterone to these cardiac changes has been extensively investigated. Initial observations indicated that aldosterone antagonists decrease left ventricular mass in patients with essential hypertension and left ventricular hypertrophy [37, 39] and improve myocardial function in hypertensive patients with diastolic heart failure [40]. However, cross-sectional evidence subsequently obtained in treatment-naïve essential hypertensive patients indicated that plasma aldosterone has no independent relationship with left ventricular diastolic properties [41]. Consistently, a recent study of hypertensive patients with diastolic dysfunction reported no change in the ventricular filling pattern after addition of spironolactone to previous antihypertensive treatment, despite a significant reduction in ventricular mass [42]. It has to be considered that lack of association between left ventricular diastolic dysfunction and plasma aldosterone levels might be related to the limitation of plasma aldosterone as a measure of the overall mineralocorticoid activity.

In 44 elderly patients with cardiac failure and preserved ejection fraction, eplerenone improved left ventricular diastolic function more than conventional treatment [43]. In the Chronic Renal Impairment in Birmingham (CRIB II) study, spironolactone improved markers of left ventricular relaxation suggesting that aldosterone blockers might be beneficial in the management of patients with diastolic heart failure [44], a hypothesis that was subsequently tested in two important trials. In the Aldo-DHF trial, spironolactone improved left ventricular diastolic function, but had no effects on maximal exercise capacity in patients with heart failure and preserved ejection fraction [45]. Similarly, in a subgroup of patients with heart failure and preserved systolic function included in the TOPCAT (Treatment of Preserved Cardiac Function Heart Failure with an Aldosterone Antagonist) trial, spironolactone significantly reduced a composite cardiovascular endpoint [46]. In summary, plasma aldosterone levels seem to be marginally relevant for left ventricular diastolic dysfunction in hypertensive subjects, but the use of aldosterone antagonists in the treatment of heart failure with preserved systolic function has so far provided encouraging results.

## 4. The Contribution of Salt to Aldosterone-Related Cardiac Damage

The hypothesis of an interplay between dietary salt and aldosterone in causing cardiac damage was extensively supported by the findings of animal studies [8, 9]. Some of the untoward effects of salt loading might depend on mineralocorticoid receptor activation resulting from changes in the intracellular redox state [47, 48]. Aldosterone affects the redox potential of diverse cell types increasing the generation of reactive oxygen species, and this effect is potentiated by exposure to high concentration of salt [49]. Therefore, an inappropriately high salt status might sensitize mineralocorticoid receptors and explain why salt interacts with aldosterone in the induction of cardiac damage.

In the clinical setting, observations on the contribution of salt to aldosterone-mediated cardiac damage are restricted to a few studies [50]. In a population study, a significant and independent correlation of the left ventricular mass index with both 24-hour urinary sodium and aldosterone excretion was reported by Jin et al. [51]. In 182 hypertensive patients who were treated for 3 years with either angiotensin-converting enzyme inhibitors or angiotensin II receptor blockers, du Cailar et al. observed a direct relationship of the percentage change in ventricular mass with the absolute changes in urinary sodium and plasma aldosterone levels [52]. In 90 essential hypertensive patients free of clinically relevant cardiovascular complications, aldosterone levels measured after intravenous saline load were found to be independently related to left ventricular mass, suggesting that limited ability of salt to modulate aldosterone production could contribute to ventricular hypertrophy [53]. In 21 patients with primary aldosteronism, Pimenta et al. found that urinary sodium independently predicts left ventricular mass [54]. More recently, we have shown that an increased left ventricular mass is associated with daily urinary sodium excretion and plasma aldosterone levels in 65 patients with primary aldosteronism [55]. Also, and most important, we found that the extent of a reduction in left ventricular mass obtained after either surgical or medical treatment of primary aldosteronism was independently correlated with the decrease in urinary sodium observed during treatment. Thus, the hypothesis of an interaction between salt and circulating aldosterone in causing damage to the heart is currently supported by robust animal data and initial clinical evidence.

## 5. Interplay of Salt and Aldosterone with Calcium Metabolism

### 5.1. Relevance to the Bone

**Figure 1:**
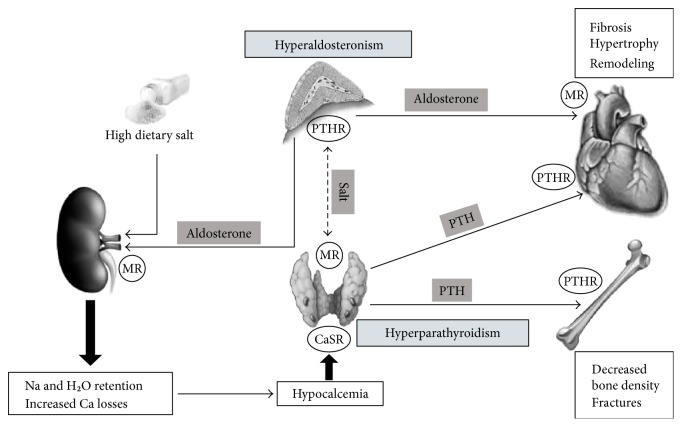
Overview of the mechanisms resulting from the interaction between aldosterone and PTH with the potential role of salt and the related impact on the heart and bone. MR: mineralocorticoid receptor; PTHR: parathyroid hormone receptor; CaSR: calcium-sensing receptor.

An interaction between components of the renin-angiotensin-aldosterone system and hormones involved in calcium homeostasis was initially suggested in patients with salt-sensitive hypertension by Resnick et al. [56] and has been reviewed in previous articles [13–15]. These authors reported for the first time a significant increase in PTH levels in patients with primary aldosteronism. Later on, a similar increase in serum PTH was demonstrated in association with increased urinary calcium excretion [57], lower serum calcium concentrations [58], and comparable vitamin D levels [59] in patients with primary aldosteronism in comparison to patients with essential hypertension. Consistent with these findings, increased prevalence of osteoporosis and increased risk of bone fracture have been reported in patients with primary aldosteronism who were recruited in different geographical areas and were compared to matched patients with essential hypertension [60, 61]. Also, and most important, normalization of serum calcium and PTH levels was reported to follow surgical treatment of patients with aldosterone-producing adenomas [59] as well as treatment with spironolactone of patients with bilateral adrenal hyperplasia [58]. These findings are in agreement with those of studies that demonstrated that administration of spironolactone to aldosterone-salt-treated rats improves cortical bone strength [62]. Consistently, treatment of primary aldosteronism was associated with significant recovery of the bone mineral density at different skeletal sites from decreased density that was detected at baseline [57]. These observations support the hypothesis that an increased fracture risk in patients with primary aldosteronism might result from secondary hyper-PTH due to aldosterone-induced hypercalciuria and subsequent hypocalcemia (Figure 1).

In support of the close interplay existing between PTH and aldosterone, recent evidence indicates that type-1 PTH receptors are expressed in aldosterone-producing adenomas [63] and explains why PTH elevation might increase aldosterone secretion. On the other hand, mineralocorticoid receptors have been detected in the nuclei of parathyroid cells indicating the possibility that aldosterone directly regulates PTH production [63]. In this context, dietary salt consumption could play an important role in as much as an inappropriate salt status causes activation of mineralocorticoid receptors leading to an increased oxidative stress and promoting tissue damage [64]. It was also suggested that salt retention and extracellular fluid expansion caused by elevated circulating aldosterone could decrease sodium reabsorption in the distal tubule leading to an increased urinary calcium excretion [65].

### 5.2. Relevance to the Heart

The interplay between salt, aldosterone, and PTH has received robust support from experimental animal studies. Treatment of rats with aldosterone and 1% dietary salt increases urinary and intestinal calcium excretion causing hypocalcemia and increased PTH secretion [66]. In these rats, blockade of mineralocorticoid receptors with the use of spironolactone decreases urinary and fecal calcium losses restoring normal calcium homeostasis [67]. The same effect of spironolactone was reported in patients with chronic heart failure [68]. When healthy subjects are exposed to a dietary salt excess, urinary calcium excretion increases significantly [69], an effect that is significantly more pronounced in patients with primary aldosteronism than in patients with essential hypertension [70]. On the other hand, many studies have shown that aldosterone causes renal calcium wasting in healthy subjects in the presence of dietary salt excess [71] and data of the Styrian Hypertension Study indicate that even in patients with essential hypertension, the interaction between aldosterone, calcium, and PTH varies depending upon the dietary salt intake [13].

In the setting of heart failure, aldosterone secretion is increased as a result of renin-angiotensin axis activation and causes salt and fluid retention. The inappropriate elevation of aldosterone for the salt status increases urinary and intestinal calcium losses with subsequent activation of PTH production [72]. This explains why elevated PTH is frequently associated with increased circulating aldosterone in patients with heart failure [73] and supports the hypothesis that PTH might concur with aldosterone in causing worsening of cardiac function in these patients [72]. Elevation of PTH levels facilitates calcium uptake in many cell types including cardiomyocytes, leading to mitochondrial calcium overload. This, in turn, decreases the ability of the cell to efficiently generate ATP thereby leading to cell death and myocardial tissue damage [70] and inducing further worsening of cardiac function [74]. In summary, both animal and human studies support the hypothesis of a functional interaction between aldosterone and PTH that could vary according to the salt status. This interaction might have an impact on bone structure and cardiac function in different disease conditions. Specifically designed studies with aldosterone blockers or other types of interventions would be needed to reach conclusive views on the pathophysiologic relevance of these mechanisms.

## 6. Conclusions

Robust scientific evidence demonstrates a relationship between salt intake and blood pressure and supports the benefits of salt restriction in hypertension. Conversely, the possible benefits of dietary salt restriction on cardiovascular outcomes are still debated because intervention studies and cumulative analyses have not been able to provide thoroughly convincing results. Current evidence unquestionably supports the view that elevated aldosterone causes cardiovascular damage well beyond what could be expected just from blood pressure elevation. Animal studies clearly indicate that aldosterone-dependent cardiac damage is strictly dependent on the salt status and this view is corroborated by the results of some human studies. A close interaction between aldosterone, calcium, and PTH has been demonstrated that is dependent on the salt status both in healthy subjects and in disease states. This complex interplay of salt with aldosterone and PTH might contribute to the development and progression of organ damage in patients with primary aldosteronism or primary hyper-PTH, in patients with heart failure, and in subjects with dietary salt excess. These observations might have important therapeutic implications inasmuch as dietary salt restriction, aldosterone blockers, calcimimetic drugs, and PTH receptor blockers might prove beneficial for cardiovascular and bone protection in these conditions. The effects of all these interventions will have to be tested in appropriately designed studies.
